# Knowledge and acceptance of recent evidence based clinical recommendations on dental caries, periodontal diseases, oral cancer and tooth wear among intern dental students. Across-sectional study

**DOI:** 10.1186/s12903-025-07207-4

**Published:** 2025-12-04

**Authors:** Nasr Mohamed Attia, Kareem Hamdi, Ahmed El-sebaai

**Affiliations:** 1 Pediatric Dentistry and Dental Public Health Department, Faculty of Dentistry, Mansoura University, Mansoura, Egypt; 2 Conservative Dentistry Department, Faculty of Dentistry, Zagazig university, Zagazig, Egypt; 3 Pediatric Dentistry Department, Faculty of Oral and Dental Medicine, Delta University for Science and Technology, Mansoura, Egypt; 4 Operative Dentistry Department, Faculty of Dentistry, Zagazig University, Al Guesh St, Mansoura City, 35516 Egypt

**Keywords:** Evidence-based dentistry, Preventive care, Intern dental students, Knowledge, Acceptance, Dental education, Egypt

## Abstract

**Background:**

Evidence-based clinical guidelines are vital for effective preventive dentistry, yet their uptake among future dentists remains uncertain.

**Objective:**

To assess and compare the knowledge and acceptance of recent evidence-based dental guidelines among intern dental students from public (Mansoura University) and private (Delta University) faculties in Egypt.

**Methods:**

A cross-sectional survey was conducted among 320 intern students using a validated Arabic questionnaire covering four domains: dental caries, periodontal disease, oral cancer, and tooth wear. Responses were rated on a 5-point Likert scale.

**Results:**

Private university students showed higher overall knowledge and acceptance scores (44.1, 43.8), while public university students exhibited slightly lower knowledge but greater acceptance in some domains (42.4, 45.3). The highest scores were seen in the periodontal disease domain. Significant differences were noted in knowledge and acceptance related to dental caries and oral cancer (*P* < 0.05). A higher proportion of “undecided” responses among public students indicated greater uncertainty.

**Conclusion:**

Both public and private university students showed moderate to high knowledge and acceptance level of evidence-based preventive guidelines. While statistically significant differences were found in some domains, effect sizes were sometimes modest with a little tendency towards private faculty students. These findings underline the need to strengthen evidence-based education across dental curricula to improve clinical readiness.

**Supplementary Information:**

The online version contains supplementary material available at 10.1186/s12903-025-07207-4.

## Background

 Evidenced-based dentistry (EBD) is a process of putting the research into practice that involve finding, evaluating, summarizing and applying the best available data to patients [1]. Emerging research is fundamentally transforming patient care, and continuing education programs alone are insufficient to keep pace [2]. Therefore, we should rely on new and updated evidence-based clinical recommendations [3]. Developed evidence-based clinical recommendations encounter assessing and criticizing the available research in a specific topic providing useful recommendations that can support clinicians in decision making. The quality of the supporting evidence is taken into consideration when grading the clinical recommendations.The strength of the recommendations and the certainty of the evidence were assessed using the GRADE approach [2, 4]. 

Several predominant clinical beliefs lack supporting evidence and are considered malpractice [5]. Accordingly, the developed evidence-based recommendations aim to equip dental students to create treatment plans that balance patient preferences, clinical experience, and current best evidence [6]. The clinical application of EBD could reduce health inequities by supporting behavior change and promoting evidence-based practices throughout life [7].

It has been speculated that a gap exists between theoretical knowledge and its practical application among dental students, which may stem from variations in educational curricula across dental schools and institutions [8]. While existing research emphasizes the importance of preventive care, there is a lack of evidence on how well dental students, particularly interns, understand and accept updated, evidence-based clinical recommendations across key oral health domains such as dental caries, periodontal diseases, oral cancer, and tooth wear. This gap is significant because effective patient care depends not only on clinical skills but also on the ability to translate current evidence into practice and to communicate preventive strategies tailored to individual patients [8]. 

To our knowledge, no studies in Egypt have compared public and private universities in relation to evidence-based awareness, leaving a gap in understanding how the educational environment influences student competencies. Therefore, this study aimed to assess and compare the knowledge and acceptance of recent evidence-based clinical recommendations among intern dental students in Egypt regarding dental caries, periodontal diseases, oral cancer, and tooth wear.

## Materials and methods

### Ethical consideration

This study was approved by the Ethical Committee of Scientific Research, Faculty of Dentistry, Delta University (Code: DU: 0240221003). All participants were informed about the study’s purpose and objectives and assured that participation was voluntary and their identities would remain confidential.

### Study design

This was a cross-sectional survey.

### Study settings

This study was conducted in both Faculties of Dentistry in Mansoura (public) and Delta (private) Universities starting from March 2024 directly after obtaining the approval of the research ethics committee. The collection of data took about 6 months.

### Eligibility criteria

Only intern dentists registered to the chosen faculties were included in the sample and all questions in the questionnaire were answered with only one answer was chosen for each question.

### Sample size calculation

The sample size was calculated to detect at least a 20% difference in the proportion of evidence-based dentistry knowledge between the study groups. This threshold was chosen based on findings by Eslamipour, F. et al. [9], who reported knowledge differences ranging from 18% to 22% among dental students. The calculation was carried out using G*Power software (version 3.1.9.4, Franz Faul, Universität Kiel, Germany), applying a two-tailed test for comparing two independent proportions, with a significance level of 0.05 and a statistical power of 0.95. Accordingly, a total of 320 participants were required, equally distributed between the two groups, to ensure adequate precision and reliability of the comparisons.

### Sampling technique

A stratified random allocation method was used to ensure equal representation of intern dental students from both public and private faculties. The total population of interns was approximately 800, with about 400 students in each faculty. A complete list of intern dental students was obtained from the intern affairs office of each faculty. Students were then assigned unique identification codes ranging from 1 to 400 for each faculty. Using a computer-generated random number application (Random.org), the required number of participants was randomly selected from each list to meet the estimated sample size. This process ensured unbiased selection and equal representation from both institutions.

### Data collection

A structured, close-ended interview questionnaire, administered in both Arabic and English, was employed to collect the necessary data (Supplementary file no.1&2). This questionnaire was prepared based on latest recommendations available from this textbook (Delivering better oral health: an evidence-based toolkit for prevention: 2021) [8] to determine the level of knowledge and acceptance of the evidence based clinical recommendations among intern dental students.

This questionnaire consisted of four parts:

- **Part I: consisted of 21 questions on dental caries** (taking different age groups with low & high risk including both behavioral recommendations and professional interventions).
- **Part II: consisted of 14 questions on periodontal disease** (taking general patients in addition to specific groups with implants, smokers, diabetics ad those taking medications that could deteriorate the gingival health and quality of saliva).
- **Part III: consisted of 6 questions on oral cancer**.
- **Part IV: consisted of 3 questions on tooth wear**.

This questionnaire was translated into Arabic language and validated regarding its validity and reliability.

A ten-member expert panel with expertise in pediatric dentistry and dental public health examined the questionnaire’s content validation. Each item was examined for translation, relevancy, and clarity. Every expert was asked to assess each item on their own and provide their thoughts and suggestions.

To determine the content validity index at the item level (I-CVI), the number of experts who rated an item as relevant or clear was divided by the total number of experts. An I-CVI above 0.79 indicated the item was appropriate; values between 0.70 and 0.79 suggested the need for revision, while items scoring below 0.70 were considered for elimination. The content validity index for the overall scale (S-CVI) was calculated using the average approach, by summing the I-CVI values for all items and dividing by the total number of items. A scale was considered valid if the S-CVI was equal to or greater than 0.90. In this study, the item-level validity indices ranged from 0.90 to 1.00, indicating excellent validity for all questions. The overall scale validity index (S-CVI) was 0.95.

After content validity, reliability was evaluated by using a pilot group of twenty intern dental students, ten of them were from Faculty of Dentistry, Mansoura University and the other ten were from Delta University. They filled out the questionnaire twice with a minimum interval of one week and they were asked to give comments concerning clarity and understanding of the questions.

Reliability was evaluated through measures of internal consistency and test-retest reliability. Internal consistency of the questionnaire was assessed using Cronbach’s alpha coefficient. The overall Cronbach’s alpha score was 0.9, and no increase in the coefficient was observed upon removal of any item. According to standard interpretation, scores below 0.5 are considered unacceptable; 0.5–0.59, poor; 0.6–0.69, questionable; 0.7–0.79, acceptable; 0.8–0.89, good; and scores of 0.9 or higher are regarded as excellent.

This questionnaire was distributed to the distinguished intern dental students by the teaching assistant staff after explaining the parts of the questionnaire to them in detail, and they were given instructions to collect data from the participants, either individually or in very small groups, and then return it to us for review.

After collecting, the questionnaires, they were evaluated for completeness, incompleteness, and answer options. If the questionnaire was not completed or more than one answer option was chosen, the questionnaire was excluded.

### Statistical analysis

IBM SPSS Statistics version 26 was used to organize, tabulate and analyze the gathered data. Qualitative data were described by using numbers and percentages on a graduated Likert scale extending from 1 to 5 (I: know this information and accept it, 2: I know this information but don’t accept it, 3: I don’t know this information but accept it, 4: I don’t know this information and don’t accept it and 5: I have no idea (I can’t make a decision).

## Results

Chi-square test was used and t-test was also used for estimating the relation between the averages of responses. The significance level was fixed at *P* ≤ 0.05. (Table 1)

**Table 1.** The average of responses

	Governmental						Private						Chi-Square (*P*-value)	Mean difference
**D.C**	1	2	3	4	5	Average	1	2	3	4	5	Average		
1	124	12	8	4	12	1.55	140	14	4	2	0	1.18	15.124 (0.004)	0.37*
2	106	16	26	4	8	1.70	132	18	8	2	0	1.25	21.154 (0.000)	0.45*
3	128	8	12	0	12	1.50	106	8	28	6	12	1.81	14.468 (0.006)	−0.31*
4	80	18	30	14	18	2.20	82	28	22	18	10	2.04	6.215 (0.184)	0.16
5	104	10	22	6	18	1.90	98	18	14	16	14	1.94	9.287 (0.054)	−0.04
6	108	22	12	8	10	1.69	112	12	16	14	6	1.69	6.222 (0.183)	0
7	118	12	16	6	8	1.59	132	10	12	0	6	1.36	7.823 (0.098)	0.23
8	140	2	14	0	4	1.29	140	12	6	0	2	1.20	11.010 (0.012)	0.09
9	148	0	6	0	6	1.23	142	8	8	0	2	1.20	10.410 (0.015)	0.03
10	130	16	12	2	0	1.29	136	10	8	4	2	1.29	4.987 (0.289)	0
11	126	4	22	4	4	1.48	128	14	8	6	4	1.40	12.505 (0.014)	0.08
12	108	12	20	6	14	1.79	112	20	16	8	4	1.58	8.358 (0.079)	0.21
13	104	2	32	10	12	1.90	132	6	18	2	2	1.35	21.718 (0.000)	0.55*
14	74	6	48	4	28	2.41	110	10	24	10	6	1.70	32.850 (0.000)	0.71*
15	154	0	4	2	0	1.09	150	2	6	2	0	1.13	2.453 (0.484)	−0.04
16	94	18	28	2	18	1.95	124	12	14	6	4	1.46	20.904 (0.000)	0.49*
17	120	6	18	6	10	1.63	130	10	12	4	4	1.39	5.571 (0.234)	0.24*
18	120	4	24	4	8	1.60	90	14	34	10	12	2	14.937 (0.005)	−0.40*
19	54	16	42	6	42	2.79	100	4	20	16	20	2.08	41.099 (0.000)	0.71*
20	124	8	16	0	12	1.55	130	8	14	8	0	1.38	20.275 (0.000)	0.17
21	138	2	12	4	4	1.34	142	2	12	2	2	1.25	1.390 (0.846)	0.09
Total	2402 (71.5%)	194 (5.8%)	424 (12.6%)	92 (2.7%)	248 (7.4%)	1.69	2568 (76.4%)	240 (7.1%)	304 (9%)	136 (4%)	112 (3.5%)	1.51	90.069 (0.000)	0.18
Total knowledge	2596 (40.1%)						2808 (42.4%)						75.484 (0.000)	
Total acceptance	2826 (43.7%)						2880 (43.5%)							
Lack of knowledge	516 (8%)						448 (6.8%)							
Lack of acceptance	286 (4.4%)						376 (5.7%)							
Undecided	248 (3.8%)						112 (1.6%)							
**P.D**														
1	138	8	4	2	8	1.34	126	22	8	4	0	1.31	17.079 (0.002)	0.03
2	154	0	6	0	0	1.08	140	18	2	0	0	1.14	20.667 (0.000)	−0.06
3	152	0	6	0	2	1.13	108	52	0	0	0	1.33	67.446 (0.000)	−0.20*
4	154	2	2	0	2	1.09	142	10	6	2	0	1.18	11.820 (0.019)	−0.09
5	144	4	10	0	2	1.20	138	8	6	6	2	1.29	8.461 (0.076)	−0.09
6	142	0	14	2	2	1.26	136	8	8	6	2	1.31	11.766 (0.019)	−0.05
7	122	2	24	6	6	1.58	126	14	10	8	2	1.41	17.115 (0.002)	0.17
8	122	6	22	8	2	1.51	142	10	6	2	0	1.18	17.258 (0.002)	0.33*
9	144	4	6	6	0	1.21	138	12	4	2	4	1.26	10.528 (0.032)	−0.05
10	94	26	14	16	10	1.89	112	26	6	12	4	1.56	7.916 (0.095)	0.33*
11	132	2	10	4	12	1.51	138	12	8	0	2	1.23	18.641 (0.001)	0.28*
12	144	6	4	2	4	1.23	138	14	6	0	2	1.21	6.394 (0.172)	0.02
13	142	8	4	4	2	1.23	134	12	12	0	2	1.28	9.032 (0.060)	−0.05
14	150	0	8	0	2	1.15	140	8	10	2	0	1.21	12.567 (0.014)	−0.06
Total	1934 (86.3%)	68 (3%)	134 (6%)	50 (2.3%)	54 (2.4%)	1.32	1858 (83%)	226 (10%)	92 (4.1%)	44 (2%)	20 (0.9%)	1.28	110.245 ((0.000)	0.04
Total knowledge	2002 (45%)						2084 (46.5%)						84.086 (0.000)	
Total acceptance	2068 (47%)						1959 (43.7%)							
Lack of knowledge	184 (4%)						145 (3.3%)							
Lack of acceptance	118 (3%)						270 (6%)							
Undecided	54 (1%)						20 (0.5%)							
**O.C**														
1	134	6	20	0	0	1.29	134	10	14	2	0	1.28	4.059 (0.255)	0.01
2	90	28	20	6	16	1.94	78	36	18	16	12	2.05	7.079 (0.132)	−0.11
3	150	0	6	2	2	1.16	144	10	6	0	0	1.14	14.122 (0.007)	0.02
4	142	0	16	0	2	1.25	142	8	8	2	0	1.19	14.667 (0.005)	0.06
5	140	2	12	0	6	1.31	138	6	12	4	0	1.26	12.014 (0.017)	0.05
6	106	4	26	0	24	1.95	138	6	12	0	4	1.29	24.040 (0.000)	0.66*
Total	762 (79.4%)	40 (4.2%)	100 (10.4%)	8 (1%)	50 (5%)	1.48	774 (80.6%)	76 (7.9%)	70 (7.3%)	24 (2.5%)	16 (1.7%)	1.37	42.075 (0.000)	0.11
Total knowledge	802 (42.9%)						850 (44.7%)						38.037 (0.000)	
Total acceptance	862 (46%)						844 (44.3%)							
Lack of knowledge	108 (5.8%)						94 (5%)							
Lack of acceptance	48 (2.6%)						100 (5.3%)							
Undecided	50 (2.7%)						16 (0.7%)							
**T.W**														
1	114	2	26	0	18	1.79	106	16	34	0	4	1.63	21.156 (0.000)	0.16
2	140	0	20	0	0	1.25	128	10	22	0	0	1.34	10.633 (0.005)	−0.09
3	148	4	6	0	2	1.15	144	8	6	2	0	1.16	5.388 (0.250)	−0.01
Total	402 (83.75%)	6 (1.25%)	52 (10.8%)	0	20 (4.2%)	1.4	378 (78.75%)	34 (7.15%)	62 (12.9%)	2 (0.4%)	4 (0.8%)	1.38	33.882 (0.000)	0.02
Total knowledge	408 (43.4%)						412 (43.1%)						33.443 (0.000)	
Total acceptance	454 (48.3%)						440 (46%)							
Lack of knowledge	52 (5.5%)						64 (6.7%)							
Lack of acceptance	6 (0.7%)						36 (3.8%)							
Undecided	20 (2.1%)						4 (0.4%)							
All	5500 (78.1%)	308 (4.4%)	710 (10.1%)	150 (2.1%)	372 (5.3%)	1.47	5578 (79.2%)	576 (8.2%)	528 (7.5%)	206 (2.9%)	152 (2.2%)	1.39	209.730 (0.000)	0.08
Total knowledge	5808 (42.4%)						6154 (44.1%)						192.706 (0.000)	
Total acceptance	6210 (45.3%)						6123 (43.8%)							
Lack of knowledge	860 (6.3%)						751 (5.4%)							
Lack of acceptance	458 (3.3%)						782 (5.6%)							
Undecided	372 (2.7%)						152 (1.1%)							

*D.C* Dental Caries, *P.D* Periodontal Disease, *O.C* Oral Cancer, *T.W* Tooth Wear

**Score 1**: know and accept; **Score 2**: know and not accept; **Score 3**: not know and accept; **Score 4**: not know and not accept; **Score 5**: no decision

**Total knowledge** (answered “know and accept” or “know and not accept”); **Total acceptance** (answered “know and accept” or “not know and accept”); **Lack of knowledge** (answered “not know and accept” or “not know and not accept”); **Lack of acceptance** (answered “know and not accept” or “not know and not accept”); **Undecided** (Unable to take decision).

A comparative analysis of the degree of knowledge and acceptance among 180 respondents from each governmental and private dental faculties regarding four major dental conditions—dental caries (DC), periodontal disease (PD), oral cancer (OC), and tooth wear (TW) was detailed # in Table 1.

The total knowledge and acceptance were consistently higher across both faculty types. However, governmental faculty respondents tended to have less knowledge and higher acceptance. This is accommodating with all the major dental conditions except for tooth wear, as the knowledge score is slightly higher in governmental faculty.

The lack of knowledge score was statistically higher in governmental faculty but represent less significantly non-acceptance pattern than private one in all dental conditions. Notably, a significant tiny proportion of respondents chose “undecided”, particularly in the governmental faculty.

Regarding dental caries, a high proportion of respondents selecting know and accept has been presented. Additionally, chi square test showed a statistical significance difference between the two faculties in different questions (*P* ≤ 0.05). Generally speaking, a governmental faculty respondents showed less knowledge and more acceptance compared to private one beside their significant inability to take decisions. There were no variations in the mean responses, according to the t-test.

Regarding periodontal disease, a very high proportion of respondents selecting know and accept has been presented. The t-test revealed no differences in the mean responses, with a propensity to both more knowledge and acceptance in private faculty respondents.

Regarding oral cancer, a significant variation was detected in the knowledge scores for ‘not know and accept’ and ‘not know and not accept’, in which it was higher in the private faculty. Also, the Chi-square test values showed significant differences in multiple questions.

Regarding tooth wear, the governmental faculty showed a significantly lower non knowledge & acceptance rate and higher instability in taking decisions. The t-test comparison indicated minimal differences without any significance.

## Discussion

Undoubtedly, harmonious integration of operator skills, theoretical knowledge, and practical experience is crucial for delivering effective dental services and treatment options. Among dental problems, carious lesions, non-carious lesions, and periodontal diseases are prevalent in population and were considered public health problems in different literature [10, 11]. A cross- sectional study was published in this regard and concluded that among 8928 patients, and the most common disease was dental caries (54.54%), followed by gingivitis (37.62%), abrasion (3.82%), malocclusion (3.05%), pericoronitis (0.53%) and jaw fracture (0.44%) [11]. Furthermore, the WHO reported in the 2022 global oral health state report that about 3.5 billion people (approximately 50% of the population) suffer from one or more of the following oral diseases: dental caries, periodontal disease, and oral cancer [12]. Economically, managing these diseases entails an annual spending of approximately US$ 387 billion in direct costs, in addition to around US$ 323 billion in indirect costs [12]. 

Furthermore, a solid foundation in EBD not only enhances students’ academic understanding but also plays a pivotal role in improving patient care and treatment outcomes. By training students to critically evaluate scientific evidence and integrate it with clinical expertise and patient preferences, EBD fosters more accurate diagnoses, effective treatment planning, and individualized care. Studies have shown that practitioners with strong EBD competencies are better equipped to choose interventions supported by current best evidence, avoid outdated or non-beneficial practices, and adjust treatment plans to align with new clinical guidelines [13]. This leads to improved preventive and therapeutic outcomes, reduced procedural errors, and enhanced patient satisfaction. Believing that knowledge and experience among intern and freshly graduated students can play a major role in preventing the spread of these diseases, and limit the burden of future treatment, we decided to conduct this cross-sectional study.

The term of knowledge and understanding EBD reflects the level of aforementioned integration of skills, knowledge, and practice for dental interns. Regarding the gap in literature between theoretical knowledge and practical applications, different published evidence emphasized the role of understanding EBD and teaching it in dental curriculum in solving this dilemma [14–16]. A cross-sectional study conducted in the European region revealed that only 32.1% of dentists reported practicing EBD, highlighting a concerning gap that warrants serious attention [17]. 

The present study demonstrated that however a significant proportion of intern dental students are aware of EBD clinical recommendations, there is variability in their acceptance and application across different domains. For instance, students demonstrated higher knowledge and acceptance levels regarding dental caries prevention strategies, aligning with the emphasis placed on caries management in dental curricula. Conversely, sections like oral cancer and tooth wear received comparatively lower scores, indicating potential gaps in education and clinical exposure. This could be attributed to the lack of implemented and taught evidence-based recommendations in the curricula of these dental institutes in this regard. Moreover, the lack of students’ exposure to corresponding cases in their institutes’ clinics may justify the available findings in this regard. In addition to these common challenges, financial constraints—such as the absence of internet access in the workplace and the unavailability of specialized textbooks in institutional libraries—may hinder students from acquiring the necessary knowledge [18, 19]. Notably, a multi-institutional study that was conducted in the United States has found that while dental students had positive attitudes towards EBD, their confidence in critical appraisal skills was limited, emphasizing the need for curriculum enhancements to seal this gap [13]. 

The findings of the present study are consistent with previous studies conducted in various regions [20, 21]. A study in Malaysia reported that However 90% of dental students exhibited a positive attitude towards EBD, only 45% practiced it regularly [20]. Similarly, research from Iran indicated that dental students possessed average knowledge and neutral attitudes towards EBD, suggesting the need for enhanced educational strategies [21]. Another study in Riyadh, Saudi Arabia, assessed the knowledge, attitudes, and practices of dental clinicians working across academic, governmental, and private sectors regarding EBD. The study demonstrated that majority of clinicians (91.8%) were aware of the term EBD. Neither the less, the last study cannot be considered a reliable reference in this case, as it involved clinicians rather than intern students [22]. In contrast, another recent cross sectional study stated that awareness of EBD was low among private dental practitioners in Saudi Arabia [23]. The last study ascribed the low level of EBD awareness to lack of time (40.9%), knowledge (22.7%), and difficulty to understand evidence-based recommendations (36.4%). Consequently, this study considered the beforementioned causes as major barriers to conduct EBD [23]. 

The observed differences in knowledge and acceptance of evidence-based guidelines between public and private dental institutions may be attributed to several underlying factors. First, private universities often have greater access to modern educational resources, including up-to-date digital libraries, simulation labs, and smaller class sizes, which can facilitate personalized learning and more frequent engagement with current guidelines. Second, the teaching faculty in private institutions may have different academic backgrounds, often with recent international training or closer ties to research activities, which may expose students to newer practices in evidence-based dentistry. Third, variations in curriculum design could also play a role; private institutions may place more emphasis on preventive and evidence-based care models as part of their competitive positioning, while public institutions might follow more traditional curricula with slower integration of recent guidelines.

Accordingly, to address the lower knowledge and acceptance scores in domains such as tooth wear and oral cancer, targeted curricular enhancements are recommended. Integrating case-based learning (CBL) modules that present real-life scenarios involving early diagnosis and prevention of oral cancer or non-carious tooth surface loss could improve clinical reasoning and guideline application. Additionally, structured problem-based learning (PBL) discussions focusing on these topics can foster deeper understanding and encourage independent learning. Incorporating interdisciplinary seminars with specialists (e.g., oral medicine, oncology, prosthodontics) may also enhance students’ exposure to advanced diagnostic tools and management protocols. Furthermore, increasing opportunities for clinical rotations in oral diagnosis units or oral medicine departments, where students can observe or participate in the examination of patients with such conditions, would bridge the gap between theory and practice.

In relation to barriers, several barriers that could impede the implementation of EBD among dental students can be acknowledged including limited access to up-to-date resources, time constraints, and insufficient training in critical appraisal skills [19, 20]. Another published evidence concluded that the main barriers for clinicians to update their knowledge was lack of equipment due to working in low-economic area [24, 25]. Without access to updated digital libraries, online databases (e.g., PubMed, Cochrane), and guideline repositories, students may rely on outdated textbooks or incomplete lecture materials and this restricts their ability to engage in self-directed learning and stay current with evolving best practices. To overcome these barriers, institutions should prioritize investment in digital infrastructure, including reliable internet connectivity and subscriptions to major EBD platforms. Strengthening university library systems by integrating open-access journals and EBD toolkits (e.g., ADA guidelines) can further support learning. Additionally, incorporating offline-compatible resources, such as mobile apps and downloadable clinical guideline summaries, could benefit students in areas with unstable internet.

Regarding the used scores in data analysis, utilizing a Likert scale from 1 to 5 enabled nuanced understanding of both the knowledge and attitudinal dimensions of participants toward clinical recommendations. The classification of responses into knowledge-acceptance categories (e.g., “know and accept”, “don’t know but accept”, “don’t know and don’t accept”, etc.) provided a varying degree of opinions and comprehensive insight into how well the students integrate theoretical knowledge with clinical attitude [26]. It is worth noting that responses of “undecided” were analyzed as a separate category and excluded from knowledge and acceptance totals to avoid bias. Missing or blank question answers were excluded.

Moreover, the questionnaire utilized in this study underwent a rigorous validation process in Arabic, employing a standardized bitranslation methodology to ascertain both linguistic and conceptual equivalence. This process entailed dual independent forward translations from the original language to Arabic, followed by backward translations through a separate set of bilingual experts. Such iterative translation cycles ensured semantic integrity and cultural appropriateness for the target demographic. To further account for potential heterogeneity in cultural and educational contexts (specifically between interns from public and private universities), a pilot study was executed on a cohort of 30 intern dental students sampled from both institutions. During this phase, comprehensive **face validity** assessments were performed, wherein participants critically appraised each questionnaire item for clarity, relevance, and contextual interpretability. This evaluative procedure aimed to confirm the instrument’s capacity to reliably capture nuanced dimensions of knowledge and acceptance concerning evidence-based dentistry recommendations. The pilot feedback substantiated the questionnaire’s psychometric robustness, revealing no requisite modifications, thereby affirming the instrument’s applicability and validity across the diverse academic settings under investigation.

The observed variations in knowledge and acceptance of evidence-based recommendations among dental interns suggest the need for targeted educational interventions within dental curricula. Specifically, the higher knowledge and acceptance scores among private university students, contrasted with the relatively greater uncertainty and “undecided” responses among public university students, indicate that institutional context may play a role in how EBD principles are absorbed and applied. To address these disparities, dental educators could incorporate structured, domain-specific learning modules—particularly focusing on areas like dental caries and oral cancer, where knowledge gaps were significant. Case-based learning, critical appraisal exercises, and journal clubs may serve to deepen understanding and promote critical thinking. Furthermore, the integration of clinical decision-making tools, such as evidence-based flowcharts and virtual simulations, can help reduce diagnostic uncertainty and improve confidence in applying current guidelines. Faculty mentorship also plays a crucial role; instructors who model EBD during patient care discussions reinforce its value in real-world practice. Collaborative educational activities, such as inter-university seminars or EBD treatment planning competitions, could also promote peer learning and standardize competencies across institutions. Ultimately, the findings underscore the importance of aligning dental education with evolving evidence, using active and context-sensitive teaching strategies to foster both knowledge acquisition and clinical acceptance of EBD.

While this study provides valuable insights into the knowledge and acceptance of recent evidence-based recommendations among dental interns, several limitations must be acknowledged. First, the self-reported nature of the questionnaire introduces a risk of social desirability bias, where students may overreport acceptance or knowledge to align with perceived academic expectations. Additionally, the cross-sectional design limits our ability to infer causality between educational background and EBD knowledge or acceptance. Differences observed between public and private university students could also be influenced by unmeasured institutional factors, such as faculty engagement with EBD, curriculum content, access to learning resources, or clinical exposure, which were not controlled for in this study. Second, the absence of long track follow-up records, longitudinal follow-up would be necessary to determine whether the observed knowledge and attitudes are retained over time or translated into clinical behavior.

## Conclusion

The implications of this study are far-reaching. Dental education institutions must prioritize updating their teaching content to align with national and international preventive recommendations. Faculty development programs focused on EBD principles, alongside curriculum revisions, may help standardize student exposure to these critical guidelines. Additionally, national accrediting bodies should mandate measurable competencies in evidence-based decision-making as a requirement for graduation. Moreover, community research should direct funding and publishing priority to research projects that addresses a real-world issue that matters to patients and clinicians, to reduce the research waste and risk of duplication repetition.

### Recommendations for enhancing EBD competencies

To foster a culture of evidence-based practice among future dental professionals, the following recommendations are proposed:

1. Curriculum Integration: Dental education programs should integrate EBD principles throughout the curriculum, ensuring that students are exposed to evidence-based decision-making processes from the early stages of their training.
2. Faculty Development: Educators should receive training in EBD to effectively mentor and guide students in applying evidence-based approaches in clinical settings.
3. Resource Accessibility: Institutions should provide students with easy access to current and relevant EBD resources, including databases, journals, and clinical guidelines.
4. Skill Development Workshops: Regular workshops and seminars focusing on critical appraisal skills, literature search strategies, and application of evidence in clinical scenarios can enhance students’ competencies.
5. Assessment and Feedback: Implementing assessments that evaluate students’ EBD skills and providing constructive feedback can reinforce learning and identify areas needing improvement.

## Supplementary Information

Supplementary Material 1

Supplementary Material 2

## Data Availability

Data is available on reasonable request from corresponding author.

## Abbreviations

EBD: Evidence Based Dentistry
I-CVI: Content validity index at the item level
S-CVI: Validity index for the overall scale
CBL: Case-based learning

## References

[1] Health Do. Delivering better oral health: an evidence based toolkit for prevention. DoH; 2007.

[2] Guyatt GH, Alonso-Coello P, Schünemann HJ, Djulbegovic B, Nothacker M, Lange S, Murad MH. Akl EAJJoce: Guideline panels should seldom make good practice statements: guidance from the GRADE Working Group. 2016, 80:3–7. 10.1016/j.jclinepi.2016.07.006 27452192

[3] Iacopino, AMJJode. The influence of new science on dental education: current concepts, trends, and models for the future. J Dent Edu. 2007;71(4):450–62. 17468305

[4] Buck DJAiaLTKsF. The English local government public health reforms. 2020.

[5] England NJUNE. New care models–vanguard sites. 2015.

[6] Clarkson J, Amaechi B, Ngo H, Bonetti DJMOS. Recall Reassessment Monit. 2009;21:188–98. 10.1159/000224223 19494686

[7] Nichol B, Wilson R, Rodrigues AM, Haighton CJCPH. A critical commentary of the Paradoxical ‘success’ of making every contact count (MECC). Crit Public Health. 2025;35(1):2518182.

[8] Godson J, Gallagher JJC. Delivering better oral health 2021–What’s new and where next? J Community Dent Health.2021;38(4):224–5. 10.1922/CDH_Dec21BDOHeditorial02 34842368

[9] Eslamipour F, Ghaiour M. Assessment of knowledge, attitude, and practice with regard to evidence-based dentistry among dental students in Isfahan university of medical sciences. J Educ Health Promot. 2016;5:12. 27500165 10.4103/2277-9531.184564 PMC4960919

[10] Tafere Y, Chanie S, Dessie T, Gedamu H. Assessment of prevalence of dental caries and the associated factors among patients attending dental clinic in Debre Tabor general hospital: a hospital-based cross-sectional study. BMC Oral Health. 2018;18(1):119. 29973262 10.1186/s12903-018-0581-8 PMC6030759

[11] Garkoti P, Rawat C, Singh RK, Rawat V, Bartwal J, Goyal NJNJMR. Pattern of dental diseases among patients attending outpatient department of dental: a hospital based cross-sectional study. J Nepal Med Res*.* 2015;5(2):112–5.

[12] Jain N, Dutt U, Radenkov I, Jain S. WHO’s global oral health status report 2022: actions, discussion and implementation. Oral Dis. 2024;30(2):73–9. 36680388 10.1111/odi.14516

[13] Straub-Morarend CL, Wankiiri-Hale CR, Blanchette DR, Lanning SK, Bekhuis T, Smith BM, et al. Evidence-based practice knowledge, perceptions, and behavior: a multi-institutional, cross-sectional study of a population of U.S. dental students. J Dent Educ. 2016;80(4):430–8. 27037451 PMC4893783

[14] Rawat P, Goswami RP, Kaur G, Vyas T, Sharma N, Singh AJJCDP. Knowledge, attitude, and behavior toward evidence-based dentistry among dental professionals in Jodhpur Rajasthan, India. J Contemp Dent Pract. 2018;19(9):1140–6. 30287718

[15] Gupta M, Bhambal A, Saxena S, Sharva V, Bansal V, Thakur B. Awareness, attitude and barriers towards evidence based dental practice amongst practicing dentists of Bhopal City. J Clin Diagn Res. 2015;9(8):Zc49–54. 26436047 10.7860/JCDR/2015/12814.6342 PMC4576641

[16] Chaturvedi A, Shah D, Gupta R. Evidence based dentistry: are we there yet? Survey of Indian dental professionals on awareness of evidence based dentistry. 2021.

[17] Yamalik N, Nemli SK, Carrilho E, Dianiskova S, Melo P, Lella A, et al. Implementation of evidence-based dentistry into practice: analysis of awareness, perceptions and attitudes of dentists in the world dental Federation-European regional organization zone. Int Dent J. 2015;65(3):127–45. 25753139 10.1111/idj.12160 PMC9376516

[18] McColl A, Smith H, White P, Field J. General practitioner’s perceptions of the route to evidence based medicine: a questionnaire survey. BMJ. 1998;316(7128):361–5. 9487174 10.1136/bmj.316.7128.361 PMC2665572

[19] Yusof ZY, Han LJ, San PP, Ramli AS. Evidence-based practice among a group of Malaysian dental practitioners. J Dent Educ. 2008;72(11):1333–42. 18981212

[20] Rath A, Wong Li Zheng M, Hesarghatta Ramamurthy P, Sidhu P, Pannuti CM, Fernandes B, et al. Evidence-based dentistry: knowledge, practice, confidence and attitude amongst Malaysian dental undergraduate students: a multi-institutional study. Eur J Dent Educ. 2023;27(1):9–18. 35023265 10.1111/eje.12770

[21] Sarani A, Sarani M, Abdar ME, Abdar ZE. Awareness, knowledge, and attitude of dentistry students in Kerman towards evidence-based dentistry. Electron Physician. 2016;8(5):2366–70. 27382446 10.19082/2366 PMC4930256

[22] ALmalki WD, Ingle N, Assery M, Alsanea JJJP, Sciences B. Dentists’ knowledge, attitude, and practice regarding evidence-based dentistry practice in Riyadh. Saudi Arabia. 2019;11(Suppl 3):S507–14. 10.4103/jpbs.JPBS_247_18 PMC6896580 31920267

[23] Gowdar IM, AlMrikhan F, Alanazi F, Alfaifi K, Alanazi B, Al-Qahishi S. Knowledge and attitude about evidence-based dental practice among dentists in Al-Kharj, Saudi Arabia. J Pharm Bioallied Sci. 2023;15(Suppl 1):S771-4. 37654258 10.4103/jpbs.jpbs_590_22 PMC10466660

[24] Castillo KB, Echeto L, Schentrup D. Barriers to dental care in a rural community. J Dent Educ. 2023;87(5):625–30. 36691321 10.1002/jdd.13176

[25] Elkady DM, Khater AGA. Knowledge and attitudes toward evidence-based cariology and restorative dentistry among Egyptian dental practitioners: a cross-sectional survey. BMC Oral Health. 2023;23(1):622. 37658399 10.1186/s12903-023-03333-z PMC10474780

[26] Koo M, Yang S-W. Likert-type scale. Encyclopedia. 2025;5(1):18.

