# Supplementary material for: Knowledge and acceptance of recent evidence based clinical recommendations on dental caries, periodontal diseases, oral cancer and tooth wear among intern dental students. Across-sectional study
Source: BMC Oral Health. 2025 Dec 4;25:1872. doi: 10.1186/s12903-025-07207-4 (PMC12676794; doi:10.1186/s12903-025-07207-4)
Supplement: Supplementary file 1 — Supplementary Material 1 [file 12903_2025_7207_MOESM1_ESM.docx]

| **تسوس الاسنان**  **كل الاطفال تحت 3 سنوات**  1. بالنسبة للرضاعة الطبيعيه - (تحدث تسوس اقل وافضل للصحة العامة)  2. - (بدون ادخال اي اطعمة حتي 6 شهور ثم ادخال الاطعمة)   1. بالنسبه للرضاعة الصناعية عن طريق البيبرونة – (تحتوي الزجاجة فقط علي لبن طبيعي او لبن اطفال او ماء بارد بعد تسخينه)   4. - (ينصح بتناول المشروبات من الكوب ابتداء من 6 شهور)  5. - (ينصح بايقافها (الزجاجة) ابتداء من سنة)   1. ينصح بادخال بعض الاطعمة الصلبةركالجزر و الكوسة ابتداء من 6 شهور بدون اضافة سكر 2. يجب علي الاباء فسيل اسنان الاطفال بمجرد بروزها مرتان يوميا (قبل النوم و مرة اخرى خلال اليوم ويجب ان يحتوي المعجون علي الف جزء من المليون فلورايد و يكون مسحة صغيرة فقط علي الفرشة بحجم حبة الرز 3. يجب ان نقلل استهلاك و عدد مرات الاطعمة و المشروبات التي تحتوي علي السكر و ايضا يفضل استخدام الادويه خالية السكر قدر الامكان و تجنب تناول المشروبات والاطعمة قبل النوم 4. يجب زيارة طبيب الاسنان كل 3 او 6 شهور علي حسب الاحتياج و خطورة التسوس القائم   **الاطفال من 3 الي 6 سنوات**   1. يجب غسيل الاسنان بواسطة الاباء وكلما كبر الطفل يجب مساعدته فقط 2. زياده حجم المعجون الي مقدار حبه البازلاء 3. عند غسيل الاسنان يقضل البصق فقط لا المضمضة حتي تتفادي تخفيف نسبة الفلورايد داخل الفم 4. يقضل اضافة دهان من الفلورايد علي الاسنان بواسطة طبيب الاسنان مرتان سنويا   **الاطفال حتي 6 سنوات اصحاب معدل التسوس العالي**   1. كل ما سبق مع زيادة تركيز المعجون الي 1350- 1500 2. تخطيط النظام الغذائي بما لا يسمح بتسوس الاسنان   **الاطفال من 7 ال 13 سنة**   1. الالتزام بغسيل الاسنان مرتين يوميا مع مساعدة الاباء عند الحاجة باستخدام المعجون بالتركيز السابق 1350- 1500 2. هؤلاء الذين لديهم معدل خطورة للاصابة بالتسوس يفضل ان يستخدمو مضمضة تحتوي علي الفلورايد فى وقت مختلف عن الفرشاة 3. يجب اضافة طلاء الفلورايد علي الاسنان الدائمة بمجرد ةظهورها مرتين سنويا 4. الاطفال 10 سنوات واكبريجب زيادة تركيز المعجون الي 2800 / 16 سنة واكثر ممكن ان يزيد الي 5000   **الكبار**   1. كل ما سبق مع تحديد مراجعة طبيب الاسنان من مره كل 3 شهور الي مرة كل 24 شهر حسب معدل خطورة التسوس 2. استخدام وسائل مساعدة اذا كان ذو قدرات خاصة   **امراض اللثة**   1. يجب ازاله البلاك من علي الاسنان يوميا بطرق مختلفة يحددها طبيب الاسنان 2. يجب ان تراعي الاتي في فرشاة الاسنان (اما يدوية او كهربائية) - (ناعمة الي متوسطة ذات راس صغيرة) 3. بالنسبة للتقويم و الكباري الثابتة (يجب استخدام ادوات خاصة للتنظيف) 4. تغيير سلوك المريض باعطائه التعليمات الازمة 5. يجب تقليل هذه الاشياء (الجير/الحشوات المعيوبة) التي تقلل من كفائة التنظيف 6. يجب مراعاة توجيه المريض اولا لاستخدام الفرشاه ثم التوجيه للادوات المناسبة لتنظيف الاسنان 7. يجب الحرص علي ان نجعل المريض يقرر اما فرشاه يدويه او كهربيه- توجيه لاختيار نوع الفرشاه و طريقة الغسيل المناسبة نلاحظ ازالة البلاك و ثقه المريض في استخدام الفرشاه و نقوم بوضع اهداف يمكن له تحقيقها في المرات القادمة   **المرضي من 12- 17 سنه مع امراض لثه او معدل خطورة عالي**   1. يجب ان يغسلو بالفرشاة تحت اللثة و يجب استخدام ادوات مناسبة ايضا لغسيل الاسنان 2. يجب ان يتواجد اهتمام طبيب الاسنان علي استخدام الوسائل المساعده للتنظيف بين الاسنان   **هولاء الذين يملكون زراعة اسنان**   1. تحتاج نفس مستوي النظافة للاسنان الطبيعية 2. المحافظة علي استخدام الفرشاة و خيط الاسناناو اي الة اخري مع الزيارة للمتابعة بانتظام   **بالنسبه للمدخنين**   1. يجب سؤالهم عن التدخين و نصحهم و العمل علي اقلاعهم عن التدخين وارشادهم للاماكن المناسبة   **بالنسبة لمرضي السكر**   1. يجب سؤالهم و العمل علي ان يظبطو مستوي السكر في الدم و عمل تحليل السكلا التراكمي للمتابعة   **بالنسبة للادوية التي تؤثر علي صحه اللثه او قله اللعاب**   1. يجب ارشاد المرضي الي خطورة تلك الادوية و يجب عليهم استشارة طبيبهم للوصول الي الحل المناسب   **سرطان الفم**   1. تظهر خطورته مع المدخنين و المدمنين علي الكحول 2. بالنسبة للمدخنين يمكن ارشادهم الي السجائر الالكترونيه الخاليه من زيت النيكوتين 3. بالنسبه للتغذية يجب زياده الخضروات والفاكهه 4. يجب الفحص المبكر (عن طريق القيام بفحص خارجي و داخلي للفم مع القيام بالتحسس) 5. يجب الغلم بان خطورة هذا المرض تزيد في تاحالات الاتيه و يجب تحويلها اذا  - قرح الفم اكثر من 3 اسابيع - وجود تورم في الشفاه او الرقبه - تغير الصوت غير مبرر - الم مستمر في الحلق عند الاكل او البلع لاكثر من 3 اسابيع  1. يجب العلم بان هناك بعض الاختبارات المناسبه للتعرف المبكر ( الصبغه او المنظار)   **تاكل الاسنان الكيميائي**   1. يجب الحفاظ علي ماسبق مع تقليل السكر و العصائر والاسموزي (لا يزيد عن 150 مل يوميا) 2. يجب ان نفحص التاكل بالاختبار المناسب 3. يجب ان نحدد اسباب لنتمكن من المعالجة بالشكل المناسب | □□□□□  □□□□□  □□□□□  □□□□□  □□□□□  □□□□□  □□□□□  □□□□□  □□□□□  □□□□□  □□□□□  □□□□□  □□□□□  □□□□□  □□□□□  □□□□□  □□□□□  □□□□□  □□□□□  □□□□□  □□□□□  □□□□□  □□□□□  □□□□□  □□□□□  □□□□□  □□□□□  □□□□□  □□□□□  □□□□□  □□□□□  □□□□□  □□□□□  □□□□□  □□□□□  □□□□□  □□□□□  □□□□□  □□□□□  □□□□□  □□□□□  □□□□□  □□□□□  □□□□□ |
| --- | --- |
