# Supplementary material for: Knowledge and acceptance of recent evidence based clinical recommendations on dental caries, periodontal diseases, oral cancer and tooth wear among intern dental students. Across-sectional study
Source: BMC Oral Health. 2025 Dec 4;25:1872. doi: 10.1186/s12903-025-07207-4 (PMC12676794; doi:10.1186/s12903-025-07207-4)
Supplement: Supplementary file 2 — Supplementary Material 2 [file 12903_2025_7207_MOESM2_ESM.docx]

| ****Tooth Decay Prevention****  ****All Children Under 3 Years Old****  1. **For breastfeeding**: Less tooth decay and better general health.  2. **No solid foods until 6 months**, then introduce solid foods.  3. **For bottle-feeding**: - Use only natural milk, formula, or cooled boiled water.    4. - Introduce drinks in a cup starting at 6 months.  5. - Stop bottle use by age 1 year.  6. Introduce some solid foods like carrots and cucumbers at 6 months, **without adding sugar**.  7- **Parents must brush their child’s teeth twice daily** (once before bed and once during the day). Use a **rice grain-sized amount** of fluoride toothpaste (1000 ppm fluoride).  8-**Reduce sugary foods/drinks** and avoid them before bedtime.  9- **Visit the dentist every 3–6 months**, depending on decay risk.  ****Children Aged 3–6 Years****  10. Parents should brush their child’s teeth, assisting as the child grows.  11. Increase toothpaste to a **pea-sized amount**.  12. After brushing, **spit out toothpaste** (no rinsing) to retain fluoride.  13. **Fluoride varnish** applied by a dentist twice yearly is recommended.  ****Children till 6 years/High-Risk Decay Groups****  14. All above, with **higher fluoride toothpaste (1350–1500 ppm)**.  15. Strict dietary guidelines to prevent decay.  ****Ages 7–18****  16. Brush twice daily with parental help if needed, using **1350–1500 ppm fluoride toothpaste**.  ****High-Risk Individuals (e.g., prone to cavities)****  17. Use **fluoride mouthwash** at a different time than brushing.  18. Apply **fluoride varnish** to new permanent teeth twice yearly.  19. For ages 10+ Increase fluoride toothpaste to 2800/ages 16+ can use **5000 ppm**  ****Adults****  20.All the above plus dental check-ups every **3–24 months**, depending on risk.  21. Special-needs individuals: Use **adaptive tools** (e.g., foam-free toothpaste, special brushes).  ****Gum Disease Prevention****  ****All Patients****  1. Remove **plaque daily** (method advised by dentist).  2. Use a **soft-to-medium, small-headed toothbrush** (manual/electric).  3. For braces/bridges: Use **special cleaning tools**.  4. Educate patients on proper oral hygiene.  5. Avoid habits that hinder cleaning (e.g., nail-biting).  6. Guide patients to use **interdental brushes/floss**.  7. Set achievable hygiene goals.  ****Ages 12–17 or High-Risk Patients**** (All the above plus)  8. Brush **under the gums** and use interdental tools.  9. Dentist must verify proper interdental cleaning.  ****Dental Implant Patients****  10. Maintain **same hygiene as natural teeth**.  11. Use brushes/floss and **regular check-ups**.  ****Smokers****:  12: Advise quitting and provide resources.  ****Diabetics**:**  13. Monitor blood sugar and HbA1c levels.  ****Medication that affect gum and saliva**:**  14. Consult a doctor if drugs harm oral health.  ****Oral Cancer****  1. High risk for **smokers/alcohol users**.  2. Suggest **nicotine-free e-cigarettes**.  3. Eat more **vegetables/fruits**.  4. **Early detection**: External/internal mouth exams.  5. **Refer if**: - Mouth ulcers last **over 3 weeks**. - Swelling in lips/neck.  - Unexplained voice changes. - Persistent throat pain/swallowing issues**.**  6. it should be noted that there are some suitable tests (e.g,toluidine blue or endoscopy)  ****Chemical Tooth Wear****  1. Maintain oral hygiene, limit sugar/juices (<150ml/day).  2. Diagnose erosion causes for targeted treatment.  3.Causes must be determined well to provide the appropriate treatment. | □□□□□  □□□□□  □□□□□  □□□□□  □□□□□  □□□□□  □□□□□  □□□□□  □□□□□  □□□□□  □□□□□  □□□□□  □□□□□  □□□□□  □□□□□  □□□□□  □□□□□  □□□□□  □□□□□  □□□□□  □□□□□  □□□□□  □□□□□  □□□□□  □□□□□  □□□□□  □□□□□  □□□□□  □□□□□  □□□□□  □□□□□  □□□□□  □□□□□  □□□□□  □□□□□  □□□□□  □□□□□  □□□□□  □□□□□  □□□□□  □□□□□  □□□□□  □□□□□ |
| --- | --- |
